# Method for positioning and rehabilitation training with the ExoAtlet ® powered exoskeleton

**DOI:** 10.1016/j.mex.2020.100849

**Published:** 2020-03-19

**Authors:** Carla Pais-Vieira, Mehrab Allahdad, João Neves-Amado, André Perrotta, Edgard Morya, Renan Moioli, Elena Shapkova, Miguel Pais-Vieira

**Affiliations:** a Center for Interdisciplinary Research in Health, Institute of Health Sciences, Universidade Católica Portuguesa, Porto, Portugal; b Centro de Investigação em Ciência e Tecnologia das Artes (CITAR), Escola da Artes, Universidade Católica Portuguesa, Porto, Portugal; c Graduate Program in Neuroengineering, Edmond and Lily Safra International Institute of Neurosciences, Santos Dumont Institute, Macaíba, Brazil; d Graduate Program in Bioinformatics, Digital Metropolis Institute, Federal University of Rio Grande do Norte, Natal, 59078-970, Brazil; e Laboratory of Neurophysiology and Technologies for Neurorehabilitation, Spinal Center of Research Institute of Phthisiopulmonology, St.Petersburg, Russia; f Laboratory of Neuroprosthetics, Institute of Translational Biomedicine, St.Petersburg State University, St.Petersburg, Russia; g Life and Health Sciences Research Institute (ICVS), School of Medicine, University of Minho, Braga 4710-057, Portugal; h Clinical Academic Center (2CA-Braga), Braga, Portugal; i iBiMED – Institute of Biomedicine, Department of Medical Sciences, University of Aveiro, Aveiro, Portugal; j Institute of Biomedical Sciences (iBiMed), Campus Universitario de Santiago, Agra do Crasto - Edificio 30, 3810-193 Aveiro, Portugal

**Keywords:** Rigid exoskeleton, Rehabilitation, Lower limb prosthetics

## Abstract

Exoskeletons for locomotion, support, or other uses are becoming more common. An increasing number of studies are demonstrating relevant results in rehabilitation. Here we describe the steps required to properly place and train patients in ExoAtlet ® powered exoskeletons (Moscow, Russia), for which there is currently limited information available. These steps combine actions related to the hardware, software, as well as safety, rehabilitation, and psycho-emotional state of the subject. Training starts with a general preparation of the environment, the equipment, and the patient. When the actual training program begins, the patient needs to gradually learn to perform the different actions that will be required to control the exoskeleton. Initially, training requires transferring weight between legs to guarantee adequate equilibrium control. Then, actions assisted by computer-controlled motors begin, namely: standing up, walking in place, moving small distances and sitting down. As the patient becomes comfortable with the exoskeleton and the cardiovascular system becomes adjusted to the upright position, training can then include walking over longer distances, inclined planes, opening doors, and climbing stairs.

• Powered exoskeletons are becoming a common method in rehabilitation.
• The use of ExoAtlet ® powered exoskeletons in clinical research requires manipulation of variables thought to promote rehabilitation, without compromising safety standards.
• The phases of training are: transferring weight between legs, walk in place, and walk over longer distances.

## Graphical abstract

**Figure:**
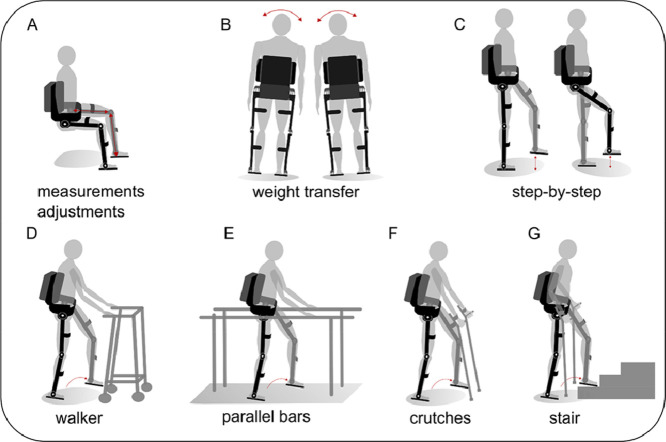

**Table:** Specifications table

Subject Area	Psychology
More specific subject area	Neuroprosthetic devices
Method name	Method for positioning and rehabilitation training with the ExoAtlet ® powered exoskeleton
Name and reference of original method	If applicable, include full bibliographic details of the main reference(s) describing the original method from which the new method was derived. Kotov, S.V., Lijdvoy, V.Y., Sekirin, A.B., Petrushanskaya, K.A. and Pismennaya, E.V., 2017. The efficacy of the exoskeleton ExoAtlet to restore walking in patients with multiple sclerosis. Zhurnal nevrologii i psikhiatrii imeni SS Korsakova, 117(10. Vyp. 2), pp.41–47
Resource availability	If applicable, include links to resources necessary to reproduce the method (e.g. data, software, hardware, reagent) N/A

## *Method details

### Materials

*Exoskeleton (with accompanying hardware, software)*

*Shoehorn*

*Flat Shoes (little or no heels)*

*Comfortable clothes (for exoskeleton user)*

*Soft measuring tape*

*Adjustable stool*

*Personnel: 1 or 2 physical therapists (or other specialized clinical staff) and one exoskeleton user*

*Parallel bars (alternative)*

*Patient lift and Harness (alternative)*

*Crutches (alternative)*

*Walker (alternative)*

*Treadmill (alternative)*

#### Steps of the method

The ExoAtlet ® powered exoskeleton can be used for clinical, social, and emotional wellbeing rehabilitation of patients with locomotor disturbances. Here we will describe how to use this device in patients with locomotor impairments (e.g., spinal cord injury, stroke, multiple sclerosis or other). As this type of training requires a large number of safety precautions, some of these steps may be skipped when a different version of the device is used. We will introduce here a sequential mix of steps involving hardware and software control, vigilance of clinical signs and symptoms, rehabilitation goals, as well as general safety measures. All of them are relevant to the rehabilitation process.

#### Eligibility for the use of exoskeleton

Before patients start training with exoskeleton, a thorough physical examination should be performed. The ExoAtlet® exoskeleton requires the user to weigh less than 100 kg (220lbs), have a height between 1.60 and 1.90 m (5.2–6.2′’), femur length between 37 and 49 cm (14.6–19.3′’), and hip width equal or less than 46 cm (18″). To guarantee that patients are able to keep balance during assisted walking, and to ensure that the patient can hold the walker or crutches (i.e. assistive device), the patient must have reasonable upper body functions, as well as a functional range of motion at shoulders, trunk, upper and lower extremities. Patients with external devices (e.g., implants, etc.) are eligible if these devices do not interfere with the ExoAtlet® use.

**Step 1 – Recharge hardware batteries**

**1.1.** Recharge tablet or laptop, and exoskeleton batteries for as long as necessary before the training. Also, check them immediately before use. The control system and the battery are located on the back of the exoskeleton. The battery takes, approximately, 6–8 h to recharge. The exoskeleton is controlled with the app on a tablet or laptop.

**Step 2 – Prepare environment, prepare patient, and prepare therapist(s)**

**Fig. 1:**
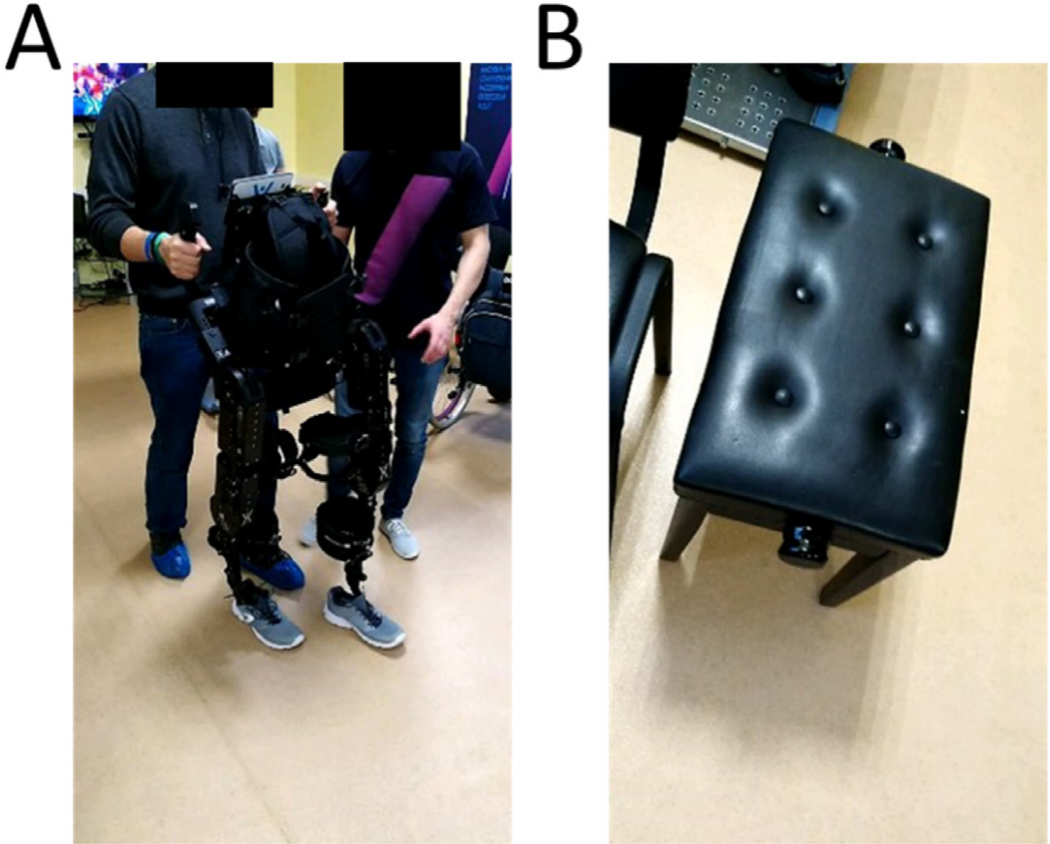
Initial training without patient in the exoskeleton – A) Example of therapist training to use the hardware and software without the patient in the exoskeleton. Note how the left foot of the therapist follows the foot of the exoskeleton to ensure that no less than three points are in contact with the floor at any given time. B) Adjustable piano stool to seat exoskeleton and transfer patients.

**2.1.** Ensure that all material is ready, and that the exoskeleton has enough room to operate. Also ensure the exoskeleton is in appropriate position to screw/unscrew before transferring the patient, adjust particular pads, etc. (Fig. 1A).

**2.2.** An adjustable piano-type chair (see Fig. 1B) is a stable platform that can be used both for exoskeleton manipulations and patient transfer.

**2.3.** If one of the therapists is not experienced with the use of the exoskeleton it is important to start by performing the exact same tasks that will later be performed with the patient, but without the patient.

**2.4.** The same procedure should also be applied in the first time that a patient is introduced to the exoskeleton (to reduce levels of anxiety both in patient and therapist).

**2.5.** As the exoskeleton will have a tight fit to the patients’ body, patients should be advised to wear light clothes and to avoid thick clothes (e.g., jeans) or clothes that have loose straps.

**2.6.** Depending on the exoskeleton model, flat shoes may also be required.

**Step 3 – Identify and measure relevant anatomical features of subject**

**Fig. 2:**
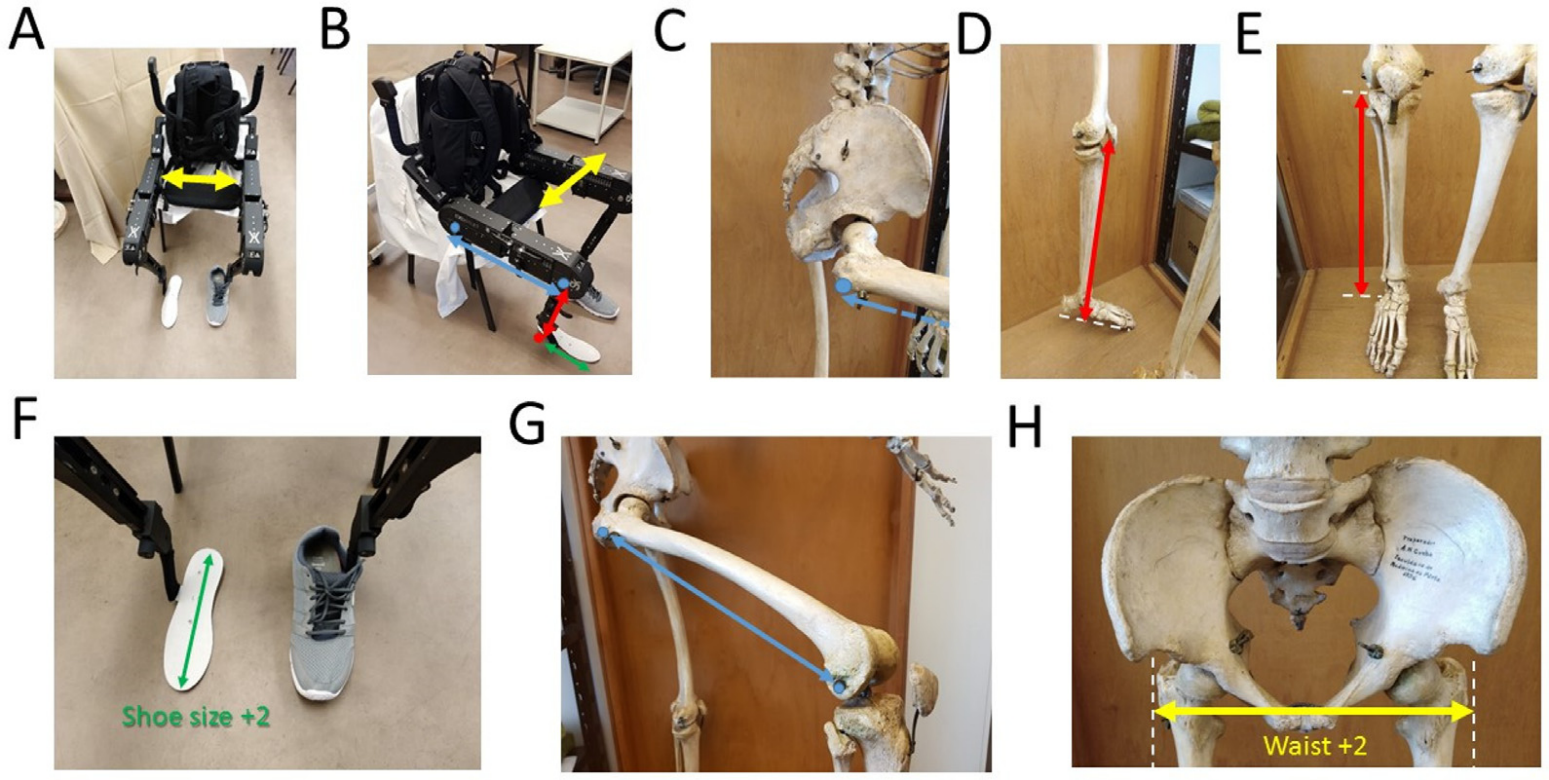
Anatomical measurements. The patient's joints should be roughly aligned to the exoskeletons’ joints. Although precise measurements may differ for different models of exoskeletons, the major principles regarding alignment should be followed. A-H, each colored arrow indicates a different measure. Arrows with the same color are shown in the exoskeleton and in the human skeleton to highlight anatomical features. The exoskeleton's shoe size should be two sizes above the patients’ shoe. Typically, two additional centimeters should be added to the patients’ waist size.

Before placing the subject/patient in the exoskeleton it is important to identify and measure specific anatomical features (Fig. 2A-H).

**3.1.** Identify subjects’ foot size (depending on the exoskeleton model this step may not be required).

**3.2.** Leg (lateral epicondyle to foot sole, when using regular shoes 1–2 cm high).

**3.3.** Thigh (greater trochanter to lateral epicondyle).

**3.4.** Waist (place hands in both greater trochanter and measure distance).

**Step 4 – Adjust exoskeleton length to subject's features**

**Fig. 3:**
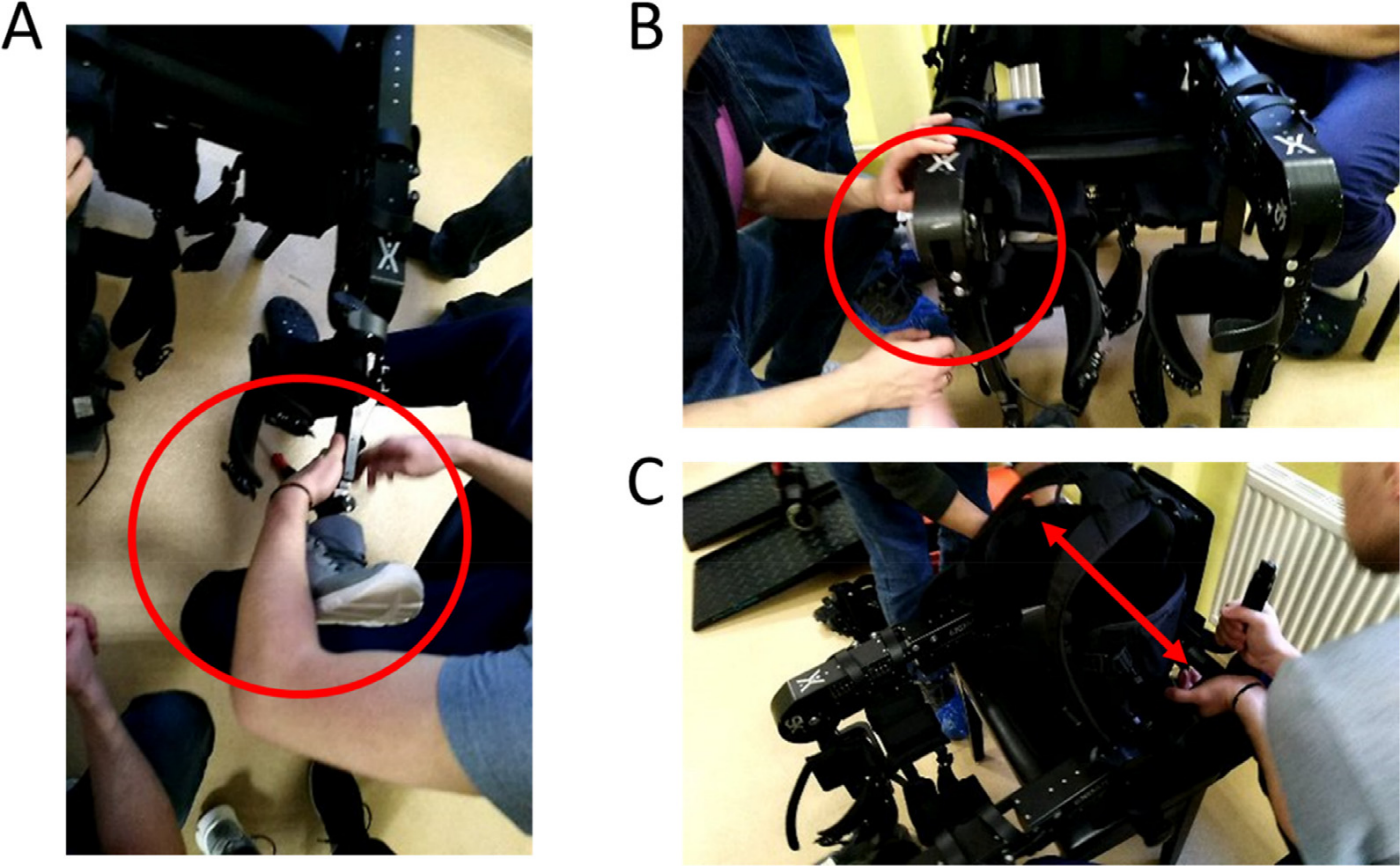
Exoskeleton measurements. Adjustments are always made from distal to proximal (i.e., from the foot towards the chest). A) Having the exoskeleton's leg supported in the therapist's own leg reduces the effort required to perform initial adjustments. B) Knee adjustments. C) Waist adjustments. Note that only one therapist makes changes in the exoskeleton at each point in time, this reduces the risk of the exoskeleton falling, or heavy movable parts becoming loose and lesion therapists.

The general rule is to start by distal locks/straps (i.e., feet) and progress to proximal locks/straps (i.e., towards chest). It is usually helpful if the therapist is with one knee in the ground, extending the exoskeleton's leg and supporting it in its’ own leg. This will reduce the effort required to make adjustments (see Fig. 3 below).

**4.1.** Check the subjects’ foot size. Recall that the exoskeleton's feet should be two sizes above the subject's size (Fig. 3A). This will ensure enough space is allowed for the feet to go in, even if there is some spasticity.

**4.2.** Adjust length of leg (Fig. 3B). As a general rule, adjustment screws should not be too tight as this may induced unnecessary stress in smaller mechanical parts.

**4.3.** Adjust thigh length. At this point you may find it necessary to also adjust the height of the adjustable stool (it is usually desirable to have the stool slightly above knee height, meaning that the exoskeleton knee angle should be somewhere between 90°−110°). This is helpful during patient transfer from the wheelchair.

**4.4.** Adjust waist size (increase two centimeters to original measurement) (Fig. 3C).

**4.5.** Ensure that environment is free of all tools (screwdrivers, non-used exoskeleton parts, etc.), place all existing straps and locks in open position (i.e., prepare exoskeleton to receive subject).

**4.6.** Turn on the exoskeleton, but do not start any training program or change position. Start by making sure the software is communicating with your particular device (i.e., there may be more than one exoskeleton in the room and software could interfere with the other devices being used). Confirm that notepad/laptop and exoskeleton batteries are still charged.

**4.7.** Introduce general parameters: patient number, therapist/physician, neurorehabilitation program, etc.

**4.8.** Having the exoskeleton turned On ensures that all joints will become locked. This is critical when transferring patients between wheelchair and the exoskeleton, because the exoskeleton will not move and can be used as an additional support by the patient.

**4.9.** Start exoskeleton program. Choose realignment function. This will ensure that the exoskeleton is properly aligned and ready to receive the patient. At this point one of the operators should be controlling the exoskeleton software and the second should ensure that exoskeleton will not be displaced from the stool.

**4.10.** Depending on the software of each exoskeleton version, this is the moment to ensure that data is being properly collected and stored for posterior analysis.

**4.11.** Check all straps and locks once again (when the exoskeleton was realigned – step 4.9 - some parts may have moved out of their initial position).

**Step 5 – Prepare patient transferring to exoskeleton**

**5.1.** The exoskeleton remains On, but no training program should be running.

**5.2.** Alternative a)

**Fig. 4:**
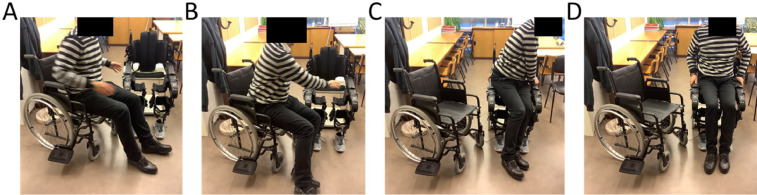
Transfer between wheelchair and exoskeleton. Transferring between the wheelchair and the exoskeleton requires locking both the wheelchair and the exoskeleton.

5.2.1. Position the wheelchair at an angle (typically between 45° to 90° degrees) to facilitate transition (Fig. 4A-B).

5.2.2. Allow patient/subject to sit in exoskeleton (Fig. 4C-D), or otherwise support transition.

**5.3.** Alternative b)

5.3.1. A harness can also be used to prevent falls. This is particularly useful for exoskeletons that do not have the option of starting from a sitting position.

**Step 6 – Don patient in exoskeleton**

**6.1.** Insert patients’ foot in exoskeleton foot (a shoehorn can be useful). Ensure that toes are in anatomical position.

**Fig. 5:**
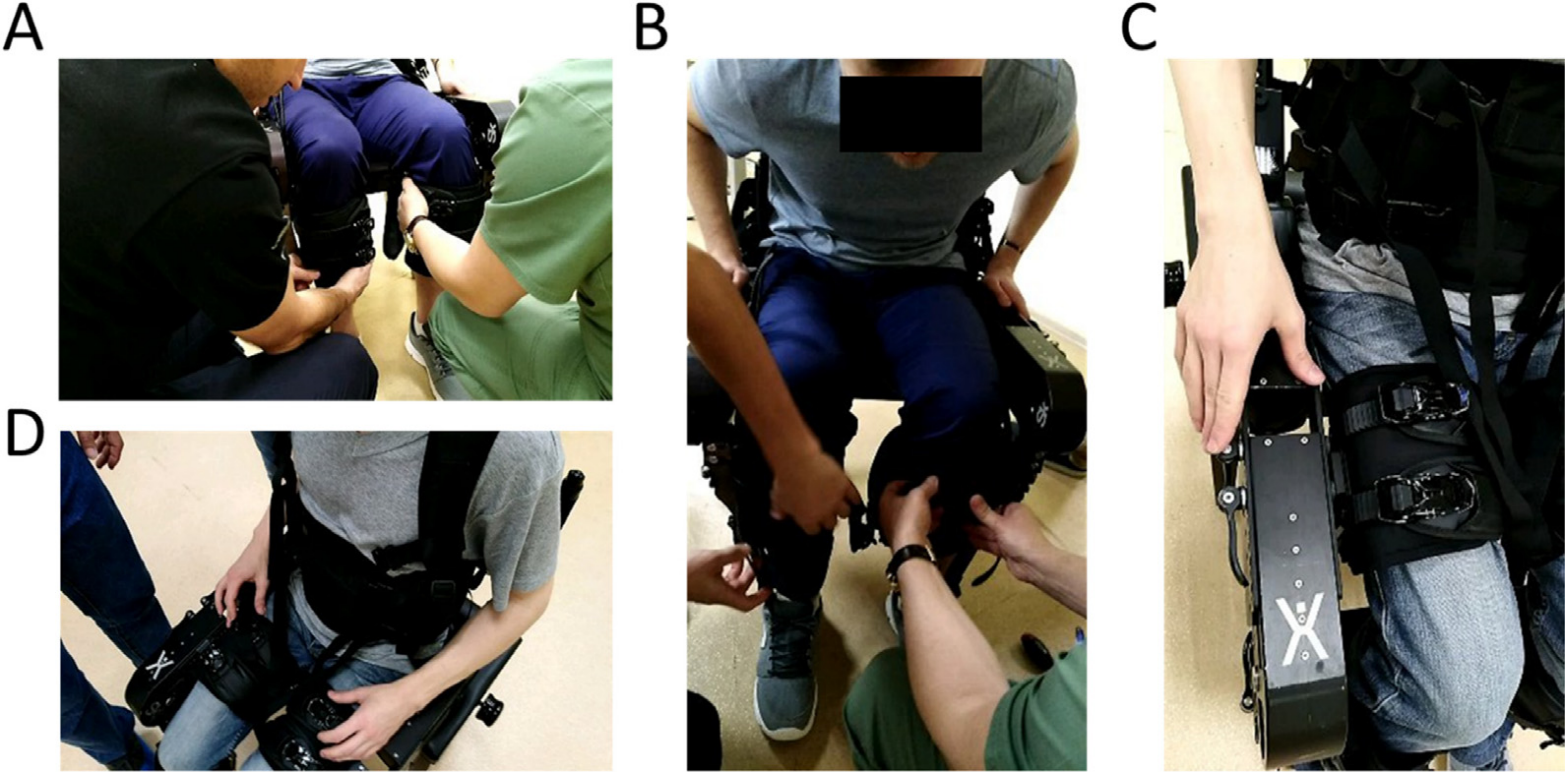
Adjusting straps in lower leg A-B and thigh C-D. When adjusting the thigh straps it is important to ensure that any existing vesical catheters do not become blocked.

**6.2.** Adjust lower strap (Fig. 5A-B). Introduce one or two protective pads, as necessary. Ensure the strap is tight (i.e., a finger can be placed between the strap and the subjects’ clothes if force is applied, but this should not be a difficult action).

**6.3.** Check thigh size support (Fig. 5C-D). The back of the thigh should be in full contact with the thigh support. Ensuring proper contact of this support structure is critical because hamstring muscles can become lesioned if this is not properly adjusted. This support should be lifted or lowered as necessary.

**6.4.** Adjust the thigh support strap. This is one of the straps where most force is exerted on the skin, so protective pads should be used. Particular care must be taken to ensure that any existing catheters or other existing medical paraphernalia are not blocked.

**6.5.** Insert arm straps, but do not tighten them yet. If the patient has some sort of damage in one arm (e.g., paresthesia), this arm should always be inserted first.

**6.6.** Adjust abdominal/chest straps. Always start with lower strap and only then move to the upper strap (this ensures proper accommodation of abdomen).

**6.7.** Tighten chest strap and adjust.

**6.8.** Make sure the patient is comfortable (if the patient is too anxious, remove patient from exoskeleton and repeat previous steps later, or in the following session).

**6.9.** Compare patients’ anatomical features with the joints of the exoskeleton (typically there will be screws in the exoskeletons’ joints). The joints of the patient and the exoskeleton should be roughly aligned (a difference up to ~2 cm can be tolerated).

**6.10.** Make sure all straps are properly tightened and loose ends are kept in safe positions or in the lateral pockets. Confirm that the patient's hands are not in contact with moving parts of the exoskeleton were they could eventually be injured. It may also be useful to refresh this information at this point.

**Step 7 – Move exoskeleton to standing or sit-to-stand position**

**Fig. 6:**
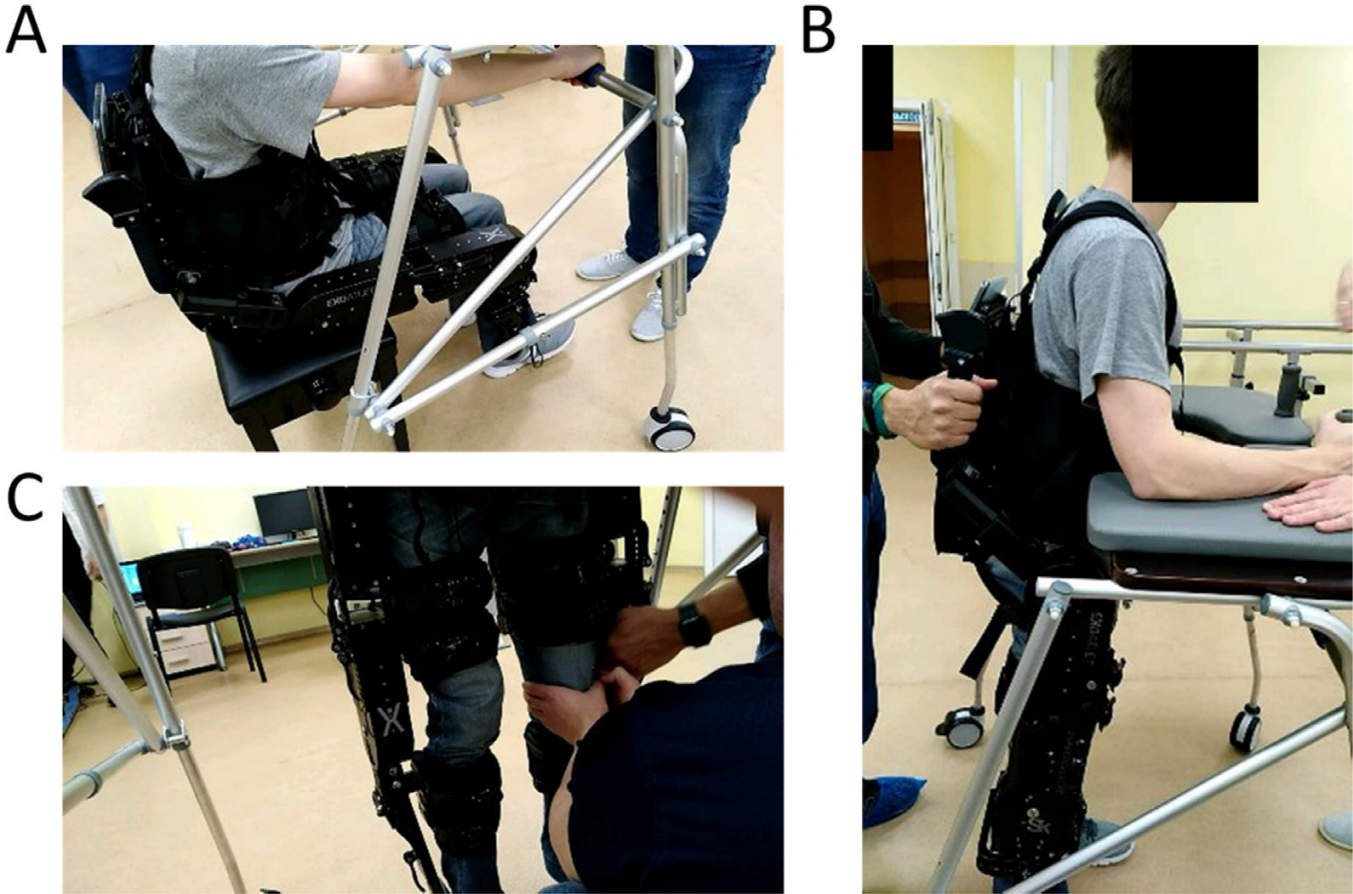
Standing up using a walker. A) Patient starts by grabbing lower bar of walker. B) One therapist stays behind the exoskeleton and another one in the front of the walker. C) When the subject stands up, small adjustments to locks and straps can be done. It is not uncommon for straps to become slightly loose when standing up.

**7.1.** Prepare the exoskeleton to stand up (Fig. 6A). This step is typically done in two parts: first, the feet are moved backwards; and second, the legs stretch as the exoskeleton rises. To prevent falls, one of the therapists should remain behind the exoskeleton and another one in the front of the exoskeleton (Fig. 6B).

**7.2.** It is important to have some form of support structure for the patient to hold on to while standing up. The exoskeleton should not be used without any support to maintain the balance. These support structures will typically be a walker, crutches, or fixed parallel bars. For patients in good physical condition, this procedure and subsequent training sessions should require the patient to use crutches independently. The handles of the crutches can also be adapted to include buttons that allow controlling the exoskeleton (i.e., smart crutches).

**7.3.** As the exoskeleton stands up, three different actions must occur: first, the patient holds on to the physical structure (for example, the walker or crutches); second, one of the therapists ensures that the patient will not fall on his/her back (recall that most exoskeletons have their batteries and microprocessors on the behind); and third, another therapist will be in the front of the patient.

**7.4.** Once the exoskeleton is in standing position, make sure the patient is comfortable. Recheck all straps and locks (see Fig. 6C). Make sure that joints are aligned and no metal part is in contact with the patient. At this point it is common for straps or locks to need some readjustment. Also, ask the patient for feedback regarding the level of comfort.

**7.5.** Evaluate cardiovascular function. Check for possible signs of hypotension or anxiety. If necessary, sit the exoskeleton and restart training in another day.

**Step 8 – Train patient to transfer weight between legs**

**Fig. 7:**
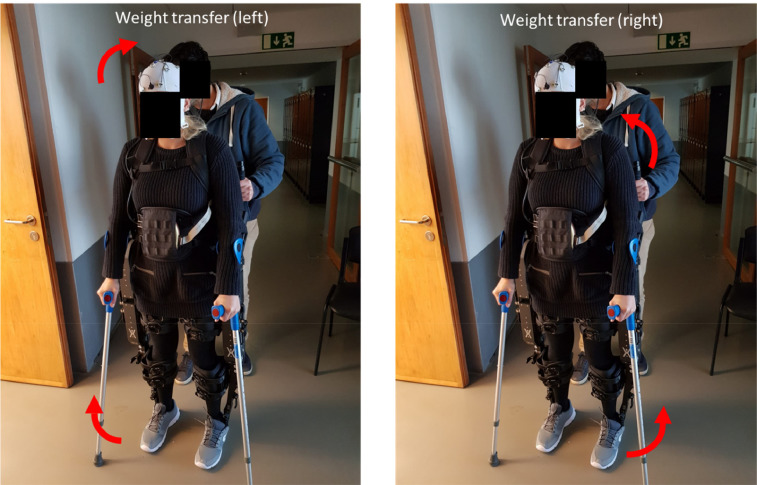
Weight transfer. Before walking, the subject needs to become familiar with the weight distribution associated with the use of the exoskeleton. For this, training starts with changing weight between the left and the right leg.

**8.1.** With the exoskeleton in standing position and before the gait-training, the patient will first, start by inclining the torso to become familiar with the new center of gravity. Then, the patient will be instructed to switch the center of gravity between the right and the left legs (standing balance procedure by Weight transfer, see Fig. 7). This can be done with both crutches and with the therapist support (e.g., the therapist that is behind the exoskeleton can help). This procedure should be performed in the first session, and for as long as necessary, until the patient reaches the goals associated with standing balance. This procedure is critical because being able to transfer the body weight from one leg to the other is the basis of the motion required to walk with the exoskeleton in all subsequent phases of training. Other exercises that can be used jointly or alternatively to this include: transferring weight laterally and posteriorly to adjust to the weight of the batteries, as well as maintaining balance for one minute using only one crutch.

**8.2.** Check if patient is using hips or shoulders to transfer weight. Hip movements are correct and will not lead to muscle soreness, while shoulder movements are incorrect and will lead to muscle soreness.

**Step 9 – Walking in place: Step-by-step**

**Fig. 8:**
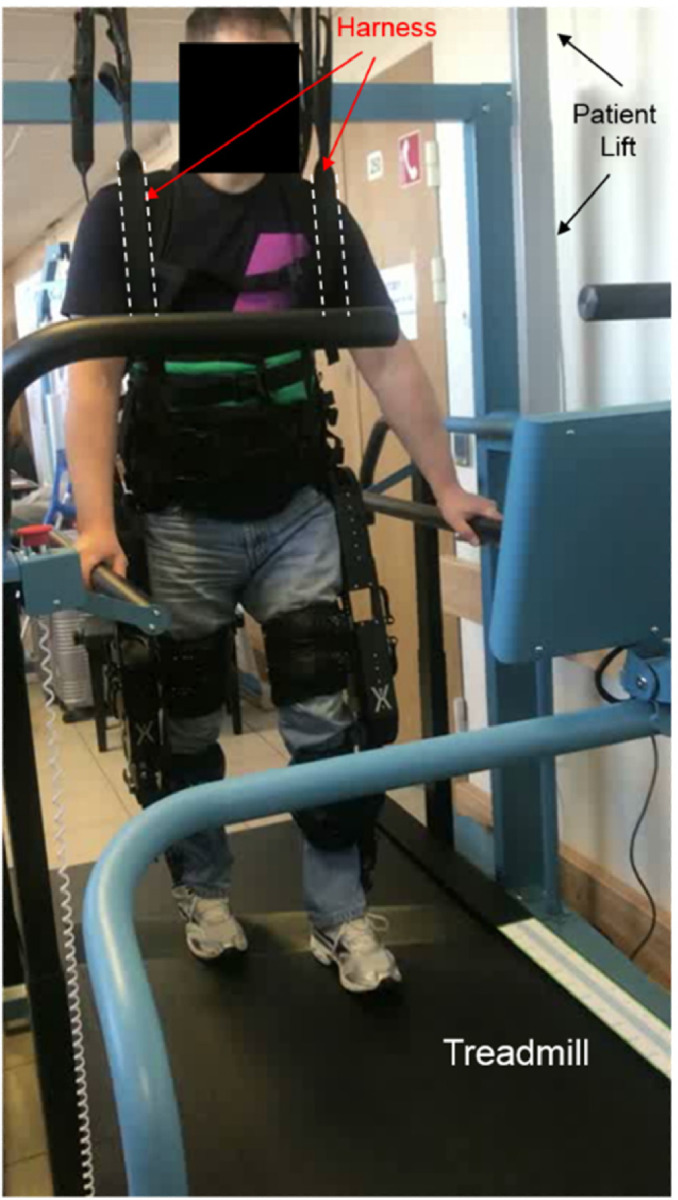
Walking in a treadmill with an exoskeleton and a safety harness.

**9.1.** In the initial phase of training, patients will typically have an increased number of additional safety measures. These can include a combination of the following: an increased number of therapists, use of the walker, crutches, parallel bars, or having the exoskeleton in a suspension system. In Fig. 8, we show an example of a suspension system for training on a safe treadmill.

**Fig. 9:**
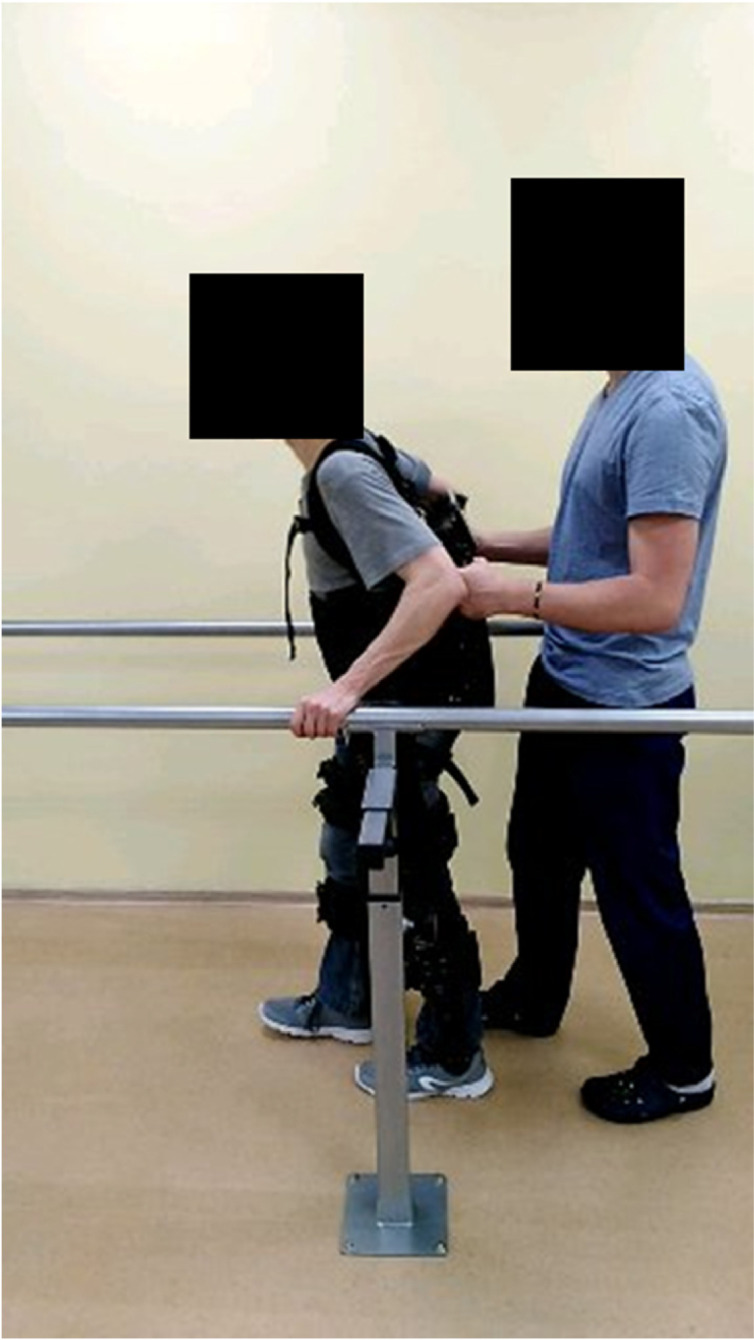
Walking with exoskeleton in parallel bars. When training with parallel bars, the arms are used only to push and never to pull the bars. The movement of pushing is critical because when patients use the crutches, they will support themselves by pushing the crutches against the floor, not pulling.

**9.2.** When patients are trained to walk (in all modalities), it is recommended that training starts with the step-by-step exercise mode, followed by the continuous step mode (with large periods of time between each step, for example 1–2 s), and lastly, ending in a continuous step exercise mode with 0 s interval between steps. This sequence of training phases (which can be modified with the introduction of intermediate phases where the length of stride or the height of the steps are changed), is required to ensure that the same sequence of movements is introduced to the patient. This will allow an increased sense of control for the patient and will ensure that joints and muscles are warmed up before walking larger distances. At this phase, patients can train, for example, outside the treadmill on parallel bars (see Fig. 9), and only then begin walking with the exoskeleton and the crutches. From our experience with patients, we have found that, if the patient's physiological condition is good and the therapists are experienced, the optimal way is to start training with crutches in the first session. This seems to lead to an increase sense of reward for the patient.

**9.3.** Always start each walking phase with the same side (for example, left leg). This way it is easier for the patient to balance when the movement starts. Before starting, always let the patient know that the exoskeleton will start to move in a period of a few seconds. At this point, it is important to avoid distracting the patient from the task at hand (e.g., talking about unrelated subjects, or commenting on previous phases of the training).

**9.4.** Warn the patient that the exoskeleton will start moving with the left leg. The therapist that is behind will start by supporting the weight transfer to the right side (to facilitate the left leg rising). Then the command to move the left leg will be pressed. As this is the first time that the patient experiences the movement of the exoskeleton while in standing position, it is important to evaluate feelings of anxiety or signs of hypotension. At this point it may also be necessary to make adjustments to the straps.

**9.5.** Warn the patient that the exoskeleton will move the right leg. Help adjust the weight for the right leg step, by tilting the weight to the left side, and proceed as in the previous step. Keep evaluating the patient's response. If no undesirable responses occur, proceed with step-by-step training until the patient indicates feeling comfortable. It may be necessary to remind the patient that weight transfer should be more related to hip movement than to shoulder movement.

**9.6.** As the patient reports being more comfortable (and if the change in weight is correctly performed with a hip movement), the interval between each step can be reduced, so that step-by-step training becomes closer to a continuous motion.

**9.7.** In the initial phases of training patients will typically be focused on the movement of their legs. It is important to remind the patient to look straight ahead while using the exoskeleton.

**Step 10 – Continuous walk**

**10.1.** Warn the patient of which leg will start moving. Also warn the patient that the movement will now be continuous. This means that neither the patient nor the therapist will give individual commands to each particular step. Instead, steps will automatically occur after a predetermined interval of time.

**10.2.** Start with longer intervals, such as 1–2 s between each step and evaluate the patient's response.

**10.3.** If no signs of anxiety or excessive cardiovascular effort are present, slowly reduce the interval between steps until a 0 s delay is used (i.e., continuous mode).

**10.4.** During walking mode, the therapist that is behind will step only after the exoskeleton steps. Walking in such a delayed mode is critical to ensure that, between the exoskeleton and the therapist, there will always be a total of 3 points of contact with the floor. When the exoskeleton is out of balance (i.e., one foot is up) the therapist should have both feet on the ground.

**Step 11 – Climbing stairs**

**Fig. 10:**
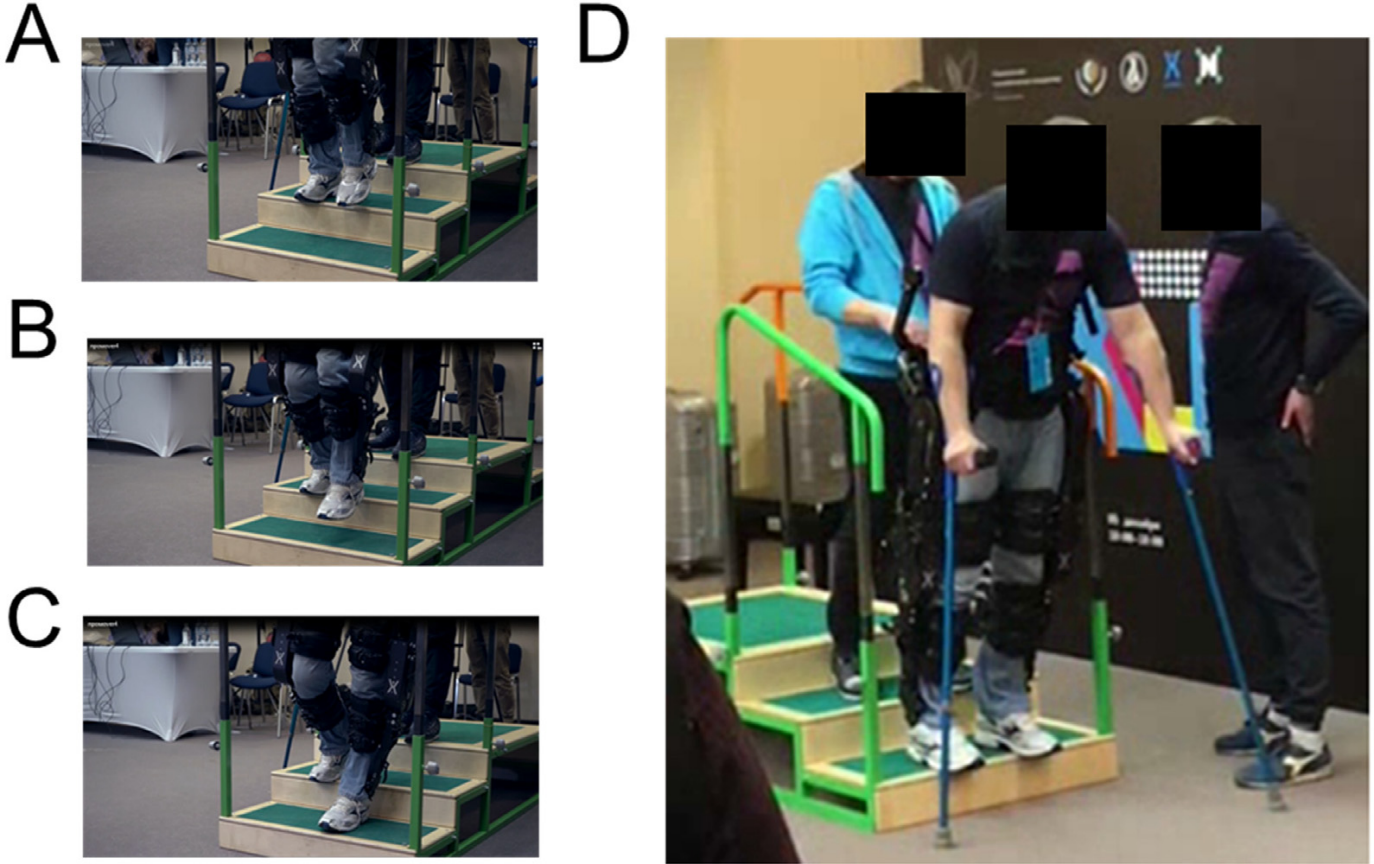
Using the exoskeleton in stairs. A-C panels show a paraplegic subject wearing the exoskeleton going downstairs. D) Overall scenario including therapist, patient, stairs, handrail, and crutches.

**11.1.** Due to the higher risk of fall, using the exoskeleton in stairs (see Fig. 10) should only be done when both patient and therapists have a high level of training in all previous steps. For safety reasons, each single stair should be considered as one individual obstacle. This means that, each stair will be climbed with both feet first, and only then another stair should be attempted.

**11.2.** Ensure that both therapists and patient are aware of the changes that will occur when the patient climbs a stair with the exoskeleton.

**11.3.** Carefully measure the height of each step and ensure that the feet of the exoskeleton can fit the step.

**11.4.** Indicate adequate parameters in software (i.e., prepare software to climb stairs).

**11.5.** Indicate to therapists and patient that climbing stairs mode will be activated. Also indicate which foot will go first.

**11.6.** Start with a step-by-step training where each stair is dealt as an individual obstacle.

**11.7.** Although it is possible, it is usually not recommended climbing stairs in a continuous mode, due to safety reasons.

**11.8.** When going downstairs the same safety measures that were described for climbing, should be followed. In addition, it should be noted that the parameters used to go upstairs may differ from the parameters used to go downstairs. This difference is related to the absence of heel movement in many exoskeletons (which make going downstairs much more dangerous than going upstairs).

**Step 12 – Sitting down and doff the exoskeleton**

**Fig. 11:**
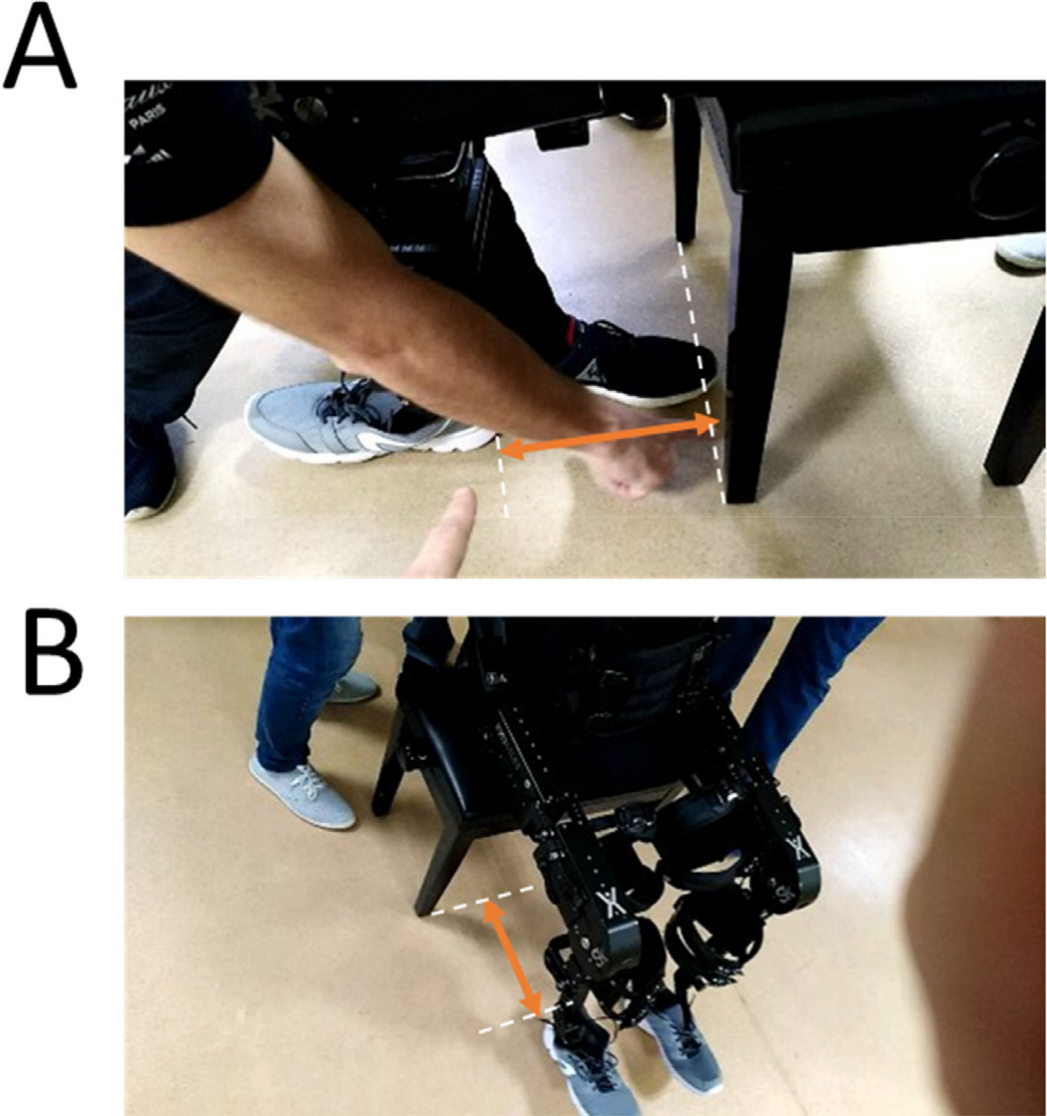
Stand to sit in the exoskeleton: A-B) From standing to sitting position. When the exoskeleton is standing, the distance required to sit is approximately one foot (red arrows).

**12.1.** For stand-sit movement transition, place the piano-type chair behind the patient to sit (see Fig. 11). This action should usually be supported by two therapists.

**12.2.** Place the exoskeleton in sitting mode in the tablet/computer controlling the exoskeleton. This action has an approximate 3-second delay.

**Step 13 – Check for signs associated with pressure injuries**

**13.1.** After training always look for possible signs and symptoms of increased pressure in the skin due to the use of the exoskeleton. Also teach the patient and/or the accompanying family on signs and symptoms that should be checked after use of the exoskeleton.

**Fig. 12:**
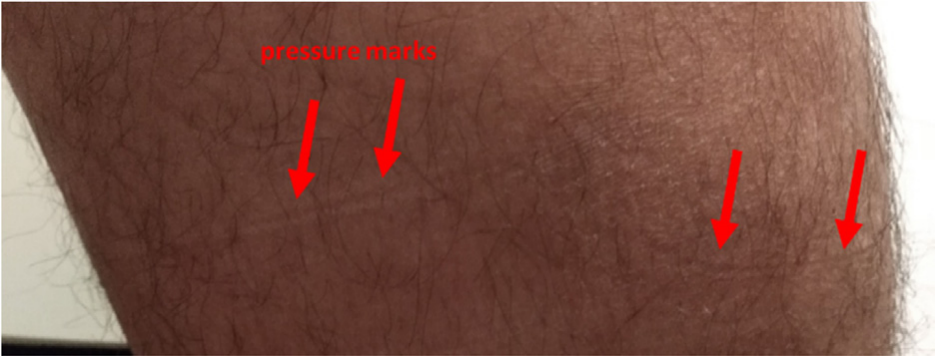
Pressure marks after exoskeleton use in a control subject. The figure shows the effect of a pressure mark associated with a thigh lock in a control subject.

**13.2.** The most common places for pressure marks or injuries are the joints and the places were locks and straps are tied (Fig. 12).

**Alternative steps**

**Alternative Step – Emergency stop**

**Fig. 13:**
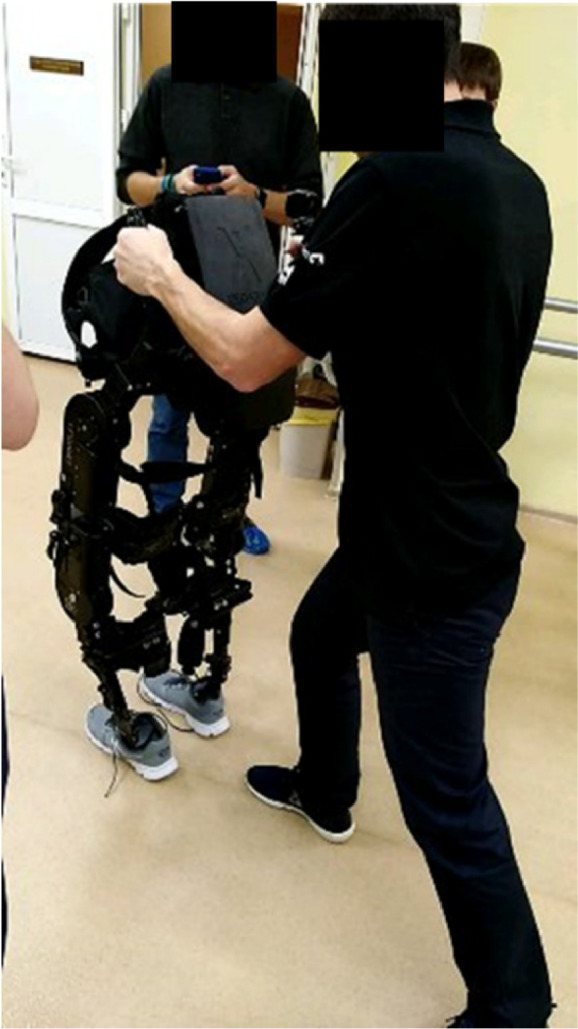
Emergency stop. If a patient requires an emergency stop, for example due to orthostatic hypotension, the exoskeleton can be turned off and put in the sitting position, so the patient can be removed. The figure shows the appropriate positioning of the therapist for a controlled descent.
• For the emergency stop, the exoskeleton can be first leaned against the therapists’ hip, turned off (to ensure that joints do not become locked) and then slowly put on sitting position on the ground. The straps can then be safely untied.
• To prevent injuries on the patient and the therapist, the therapist can use the hip to support the exoskeleton and then slowly drop the exoskeleton to sitting position.

• The need for an emergency stop should always be considered when using exoskeletons (Fig. 13). This could be due to problems related to the patient, to the exoskeleton hardware or software, to the therapist, or the environment.

**Alternative Step – Muscle spasm or voluntary movement against exoskeleton**

• If the patient has a muscle spasm or, alternatively, forces the exoskeleton to perform a particular action, this will be detected by force sensors in most exoskeletons. The software and hardware are prepared to detect this event and stop the running program. However, it is important for therapists to support the exoskeleton with the patient (also see Alternative step Emergency Stop). The program can then be restarted or, otherwise, the exoskeleton with the patient can be put in sitting position on the floor and the straps loosen.

**Alternative Step – Perform EEG recordings during exoskeleton use**

**Fig. 14:**
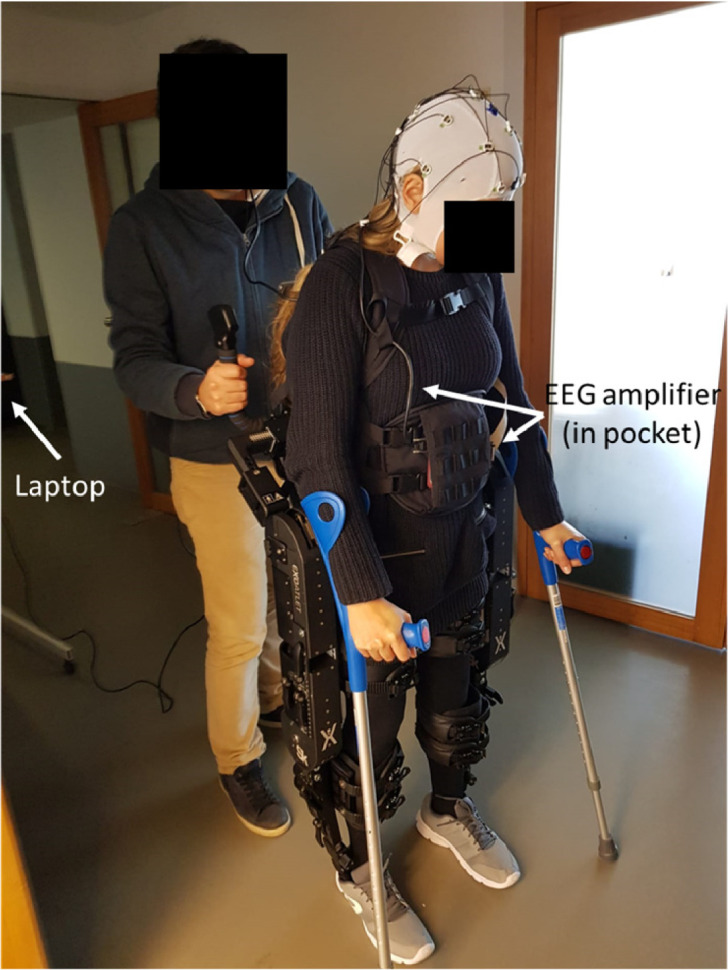
EEG recordings during exoskeleton use. When recording EEG activity with the exoskeleton it is useful to have the laptop using its own battery (i.e., not using the power plug) and distant from the exoskeleton battery.
• As the EEG is an additional technique that can both take additional time and often requires the use of conducting gel, it is recommended that the EEG cap and electrodes are placed after the patient has already done a small test in the exoskeleton (for example, taken a couple of steps in the same place). This will reduce the chances of having to stop the recording due to postural hypotension, discomfort with exoskeleton, etc.
• Start by sitting the patient, and only then place EEG cap and gel. As these procedures can take 15–30 min depending on the amount of channels recorded, always ensure that the patient is sitting during this period.

• In some cases, it may be useful to record neuronal signals while using the exoskeleton (for example, to study neural activity associated with gait, or during use of brain-machine interfaces) [2] (Fig. 14).

To summarize the information described above, Table 1 presents a summary of the steps, main actions, goals, duration, evaluation as well as periodicity and additional notes.

**Table 1:** Summary of steps, goals, and expected duration.

Step	Main actions	Goal	Duration	Evaluation	Periodicity and notes
1	Check batteries	Safety	1 min	Batteries charged	Every session
2	Prepare environment	Safety	1 min	Enough room for walking, using tools, weelchair, etc.	Every session
3	Measure anatomical features	Exoskeleton Fit	5–10 min	Joints are aligned to exoskeletons' joints	First session only
4	Adjust exoskeleton measures	Exoskeleton Fit	5–10 min	Joints are aligned to exoskeletons' joints	Every session
5	Transferring patient to exoskeleton	Safety	1 min	No falls	Every session
6	Don patient in exoskeleton	Safety	5–10 min	Joints are aligned to exoskeletons' joints. Patient is confortable	Two/three times per session
7	Move exoskeleton to standing position	Neurorehabilitation	1 min	Joints are aligned to exoskeletons' joints. Patient is confortable No falls. No cardiovascular signs or symptoms	Two/three times per session
8	Train patient to transfer weight between legs	Neurorehabilitation/Safety	1–5 min	No falls. No cardiovascular signs or symptoms	Mostly first session, repeat if necessary
9	Walking in place: step-by-step	Neurorehabilitation/Safety	1–5 min	Patient is confortable. No falls. No cardiovascular signs/symptoms	Every session (if trained)
10	Continuous walk	Neurorehabilitation/Safety	variable	Patient is confortable. No falls. No cardiovascular signs/symptoms	Every session (if trained)
11	Climbing stairs	Neurorehabilitation/Safety	variable	Patient is confortable. No falls. No cardiovascular signs/symptoms	Every session (if trained)
12	Sitting down	Safety	1 min	Patient is confortable. No falls. No cardiovascular signs/symptoms	Every session (if sitting)
13	Evaluate effects of Exoskeleton on skin	Safety	1 min	No pressure marks.	Every session Inform family/ patient on signs/ symptoms

## Method validation

To demonstrate the different steps of the method described above, we present below: 1) the results from a control subject throughout the initial three sessions with an exoskeleton, 2) the Electroencephalography (EEG) topographic maps of this control subject recorded during the last session, and 3) the results from a spinal cord injury patient trained with the exoskeleton. The present experiments were approved by the Ethics Committee of the St.Petersburg State Research Institute of Phthisiopulmonology (protocol 28/2017).

### Method validation I – Exoskeleton use and EEG recordings in a control subject

**Fig. 15:**
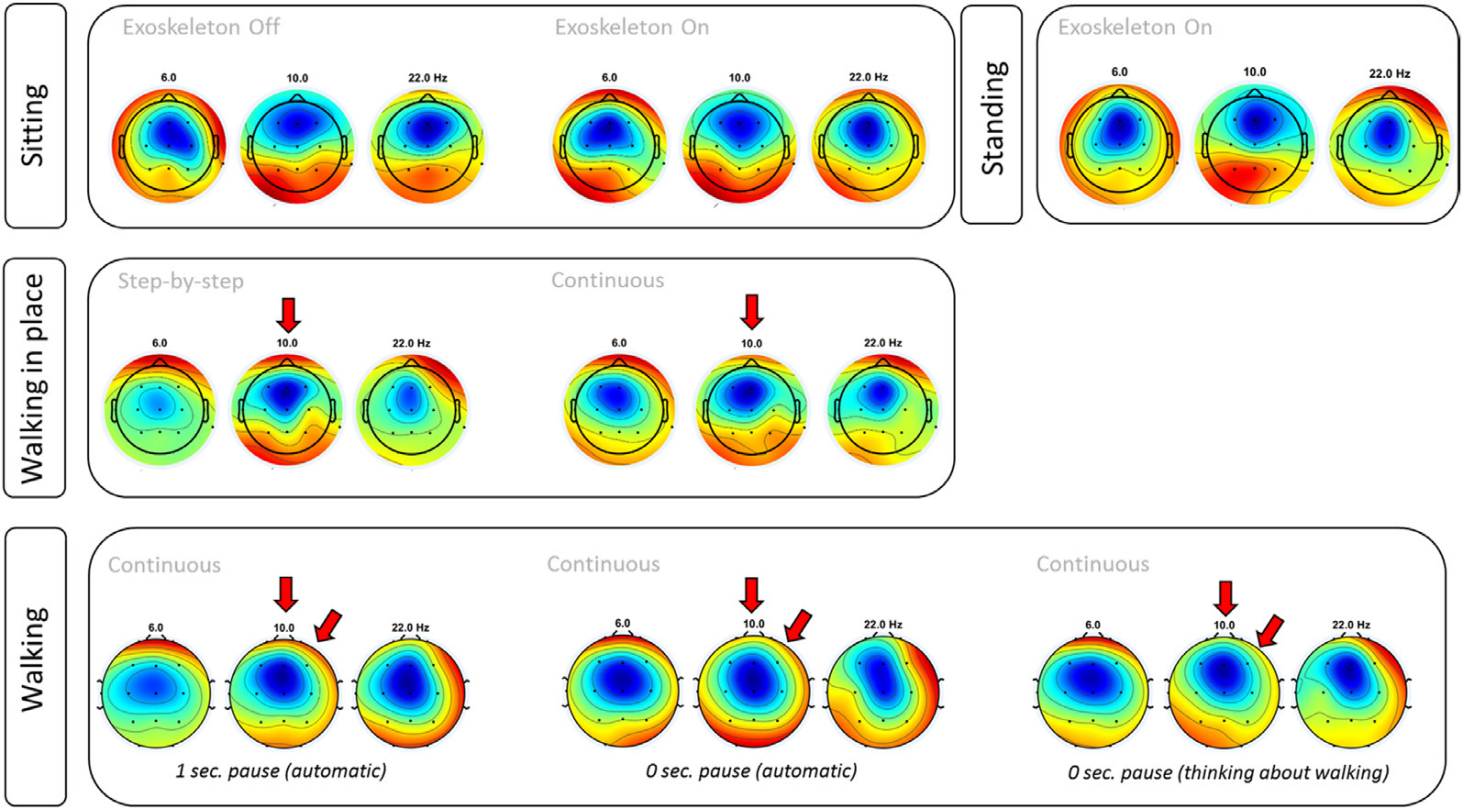
EEG activity during exoskeleton use. *Step-by-step* or *continuous* modes indicate if the start of each step was generated by the therapist or automatically by the software. The top row shows EEG activity recorded with the subject Sitting and the exoskeleton OFF (left), ON (center) and ON with the subject standing (right). When the subject was walking in place (Center panels) an increase in neural activity was observed at 10 Hz (red arrows, center). When the subject was walking (lower panels), an increase in power was observed also at 10 Hz (red arrows, bottom).

To demonstrate the different steps of the method described above, we present here the results from a control subject learning to use the exoskeleton (Table 2), as well as the EEG results from brain activity recorded during the last session (Fig. 15).

**Table 2:** Evolution of control subject in the use of an exoskeleton. In the first session the subject was anxious and revealed a high level of discomfort. Training had to be discontinued. During the second and third sessions the subject reported a reduction in levels of anxiety and discomfort, while most of the other measures improved.

	Session number		
	#1	#2	#3
Standing position (mins)	5	15	20
Weight transfer	Yes	Yes	Yes
Walk in place (number steps)	5	10	10
Walk (number steps)	0	30	50
Anxiety, self-reported (1–10)	10	8	5
Discomfort, self-reported (1–10)	10	6	5
Support besides therapist behind	Therapist in front	Therapist in front	Crutches
Other monitoring techniques	No	No	EEG recording

During the first session, the subject was only trained to change weight between the right and left legs, and to walk in place. This subject reported high levels of anxiety and discomfort and thus, training was interrupted due to discomfort.

During the second session (performed in the same day), the subject reported lower levels of anxiety and discomfort. In this session the subject was able to transfer weight between right and left legs, to walk in place, and to walk for a relatively small distance with two therapists supporting (one in the front and another one behind). Lastly, in the third session, lower levels of anxiety were reported by this subject. These lower levels of anxiety were accompanied by: longer distances travelled with the exoskeleton, being able to use crutches, and requiring only one therapist for walking exercises.

Analysis of neural activity recorded during the third session suggested that the exoskeleton batteries did not interfere with the recorded signal (compare topographic map “Exoskeleton Off” with topographic map “Exoskeleton On”) and also indicated that activity related to Walking was mostly associated with increases in power close to the 10 Hz frequency band (see red arrows).

### Method validation II – Exoskeleton use in a spinal cord lesion patient

**Fig. 16:**
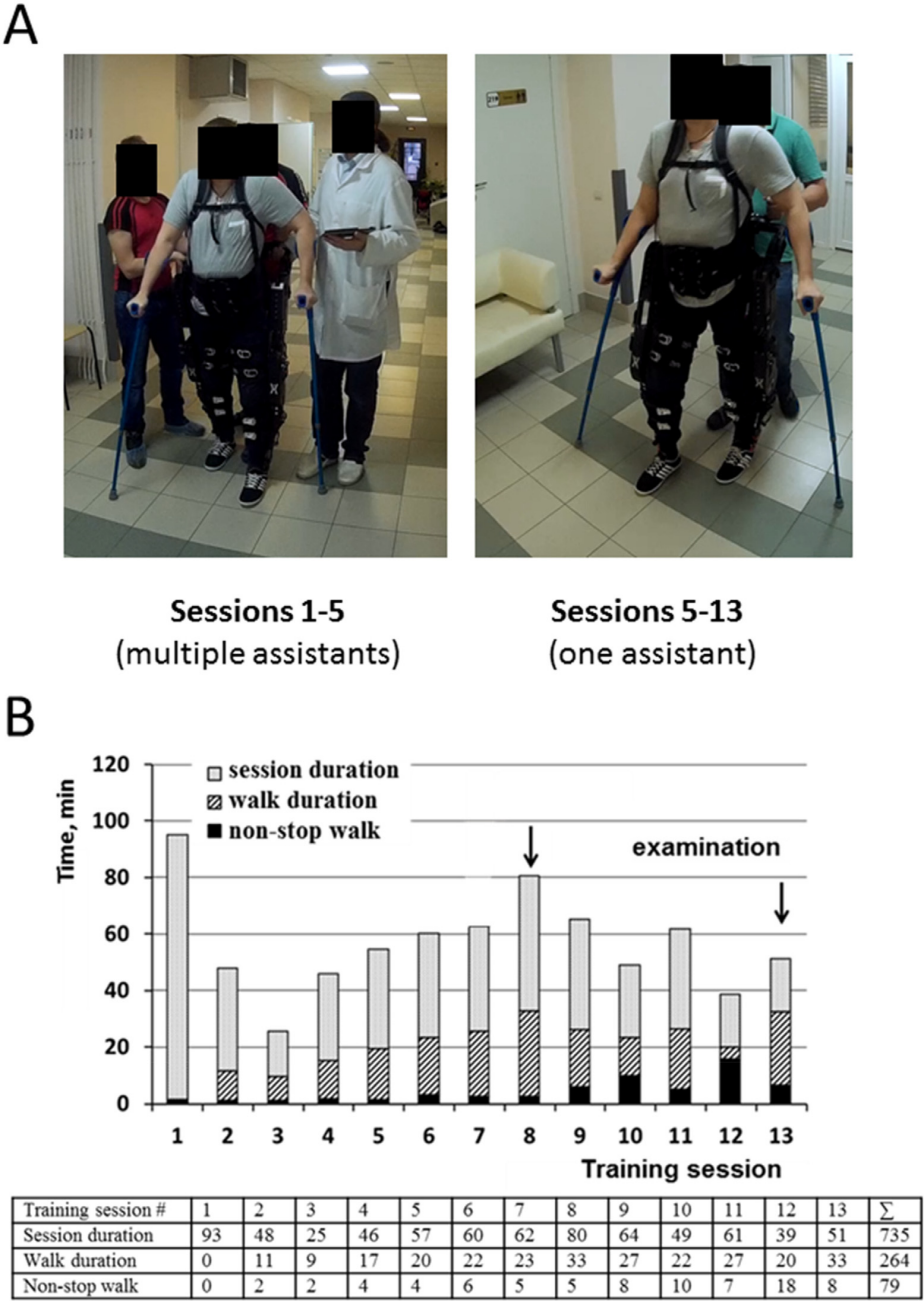
Spinal cord lesion patient trained with ExoAtlet® powered exoskeleton. The figure shows the evolution of several parameters throughout 13 sessions where a spinal cord injury patient used the exoskeleton. The arrows indicate sessions 8 and 13, which are further detailed in Fig. 17.

As exoskeletons are more often used for rehabilitation, we also present here the results from one spinal cord injury patient training with the ExoAtlet® exoskeleton (Fig. 16). This patient was a 21 year-old male with a fracture of Th5-7, and spinal cord damage D4. Clinical evaluation at the beginning of training was ASIA A, Motor – 1; Sensory Light Touch-40, Pin Prick- 43, Rivermead Mobility Index −0; and Bartel Index 25. Exoskeleton training started 2 years post-trauma, results from the first 13 sessions are presented here. As the main goal of the present manuscript is not the clinical evaluation we will not present further details related to the neurological evaluation. In Fig. 16 several parameters related to the use of the exoskeleton are presented. Initially this patient required two therapists (Fig. 16A), but throughout the course of multiple sessions, this patient ended requiring only one therapist. Also, parameters such as: walk duration and amount of time with non-stop walking, increased throughout the course of multiple sessions (Fig. 16B).

**Fig. 17:**
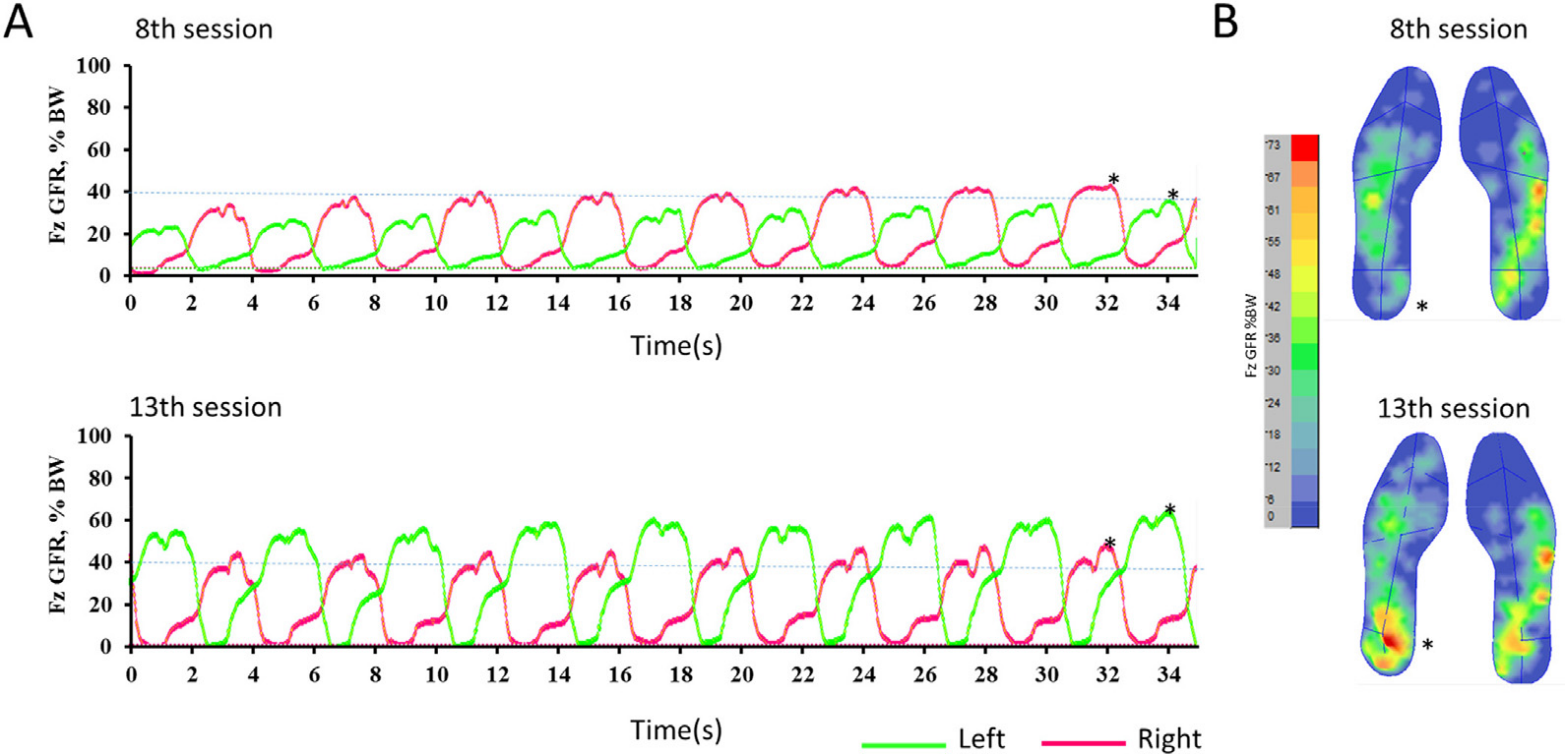
Evolution of sole support area throughout training. Fz GFR%BW indicates the ground reaction force as percent of body weight. A) The dashed line indicates 40% support in both sessions, to allow comparison of evolution. The asterisks in A indicate which left and right steps are represented in B. As training progressed an increased area of support related to the calcaneus was observed.

A more detailed analysis of the amount of body weight that was supported by the sole of the foot suggested and overall increase as training progressed. In the example presented in Fig. 17A and B, an overall increase in the area supported was observed, especially in the left calcaneus.

## Declaration of Competing Interest

The authors declare that they have no known competing financial interests or personal relationships that could have appeared to influence the work reported in this paper.
